# Glycogen, poly(3-hydroxybutyrate) and pigment accumulation in three *Synechocystis* strains when exposed to a stepwise increasing salt stress

**DOI:** 10.1007/s10811-022-02693-3

**Published:** 2022-03-30

**Authors:** K. Meixner, C. Daffert, D. Dalnodar, K. Mrázová, K. Hrubanová, V. Krzyzanek, J. Nebesarova, O. Samek, Z. Šedrlová, E. Slaninova, P. Sedláček, S. Obruča, I. Fritz

**Affiliations:** 1 Institute of Environmental Biotechnology, Department of Agrobiotechnology, IFA-Tulln, University of Natural Resources and Life Sciences, Vienna, Konrad-Lorenz-Straße 20, 3430 Tulln, Austria; 2 BEST Bioenergy and Sustainable Technologies GmbH, Inffeldgasse 21b, 8010 Graz, Austria; 3 Institute of Scientific Instruments, The Czech Academy of Sciences, Královopolská 147, 61264 Brno, Czech Republic; 4 Institute of Parasitology, Biology Centre, The Czech Academy of Sciences, Branisovska 31, 37005 Ceske Budejovice, Czech Republic; 5 Faculty of Science, Charles University, Vinicna 7, 128 44 Prague 2, Czech Republic; 6 Faculty of Chemistry, Brno University of Technology, Purkynova 118, 612 00 Brno, Czech Republic

**Keywords:** Salt stress, *Synechocystis* sp., Poly(3-hydroxybutyrate), Glycogen, Pigments

## Abstract

The cyanobacterial genus *Synechocystis* is of particular interest to science and industry because of its efficient phototrophic metabolism, its accumulation of the polymer poly(3-hydroxybutyrate) (PHB) and its ability to withstand or adapt to adverse growing conditions. One such condition is the increased salinity that can be caused by recycled or brackish water used in cultivation. While overall reduced growth is expected in response to salt stress, other metabolic responses relevant to the efficiency of phototrophic production of biomass or PHB (or both) have been experimentally observed in three *Synechocystis* strains at stepwise increasing salt concentrations. In response to recent reports on metabolic strategies to increase stress tolerance of heterotrophic and phototrophic bacteria, we focused particularly on the stress-induced response of *Synechocystis* strains in terms of PHB, glycogen and photoactive pigment dynamics. Of the three strains studied, the strain *Synechocystis* cf. *salina* CCALA192 proved to be the most tolerant to salt stress. In addition, this strain showed the highest PHB accumulation. All the three strains accumulated more PHB with increasing salinity, to the point where their photosystems were strongly inhibited and they could no longer produce enough energy to synthesize more PHB.

## Introduction

Cyanobacteria have a long evolutionary history in which they colonized a wide variety of habitats, ranging from glaciers to hot springs, from freshwater to saline environments. They evolved different strategies to cope with environmental stress and they manage to adapt to changing environmental conditions within their habitats. Cyanobacteria are capable of acclimating to various stress conditions, by adapting their physiological, biochemical and molecular activities. They respond to temperature stress by altering membrane composition to adapt fluidity through saturation of fatty acids and protein integrity. Strategies used in response to UV stress include scavenging reactive oxygen species, synthesis of UV-absorbing/shielding compounds, repair of damaged DNA and resynthesis of proteins. Against metal-induced stress, cyanobacteria protect themselves by altering their membrane structure and activating or inactivating transfer pumps. Besides that, salt stress is a major abiotic stress problem in arid and semi-arid regions as well as in irrigation areas (Sudhir and Murthy 2004). Salinity reduces the availability of water and increases the Na^+^ and Cl^−^ concentration. Cyanobacteria reduce the Na^+^-uptake as well as their active efflux via Na^+^/H^+^ antiporter, they induce organic compounds, increase antioxidative defense systems to detoxify the reactive oxygen species, accumulate compatible solutes to compensate osmotic pressure and finally, also express salt-inducible proteins to cope with salt stress (Klähn and Hagemann 2011; Rezayian et al. 2019).

Besides these survival strategies, cyanobacteria, such as *Synechocystis* sp., or heterotrophic bacteria, such as *Cupriavidus necator* accumulate carbon and energy storage polymers. It is known that the lipophilic poly(3-hydroxybutyrate) (polyhydroxybutyrate, PHB) is formed under nitrogen and/or phosphorus deficiency with simultaneous excess of carbon and energy. PHB is known to act in heterotrophic bacteria as a protectant against osmotic and oxidative stress, against pH fluctuations and increased pressure (Obruca et al. 2020, 2021; Fritz et al. 2021). Besides PHB, cyanobacteria produce the hydrophilic polymer glycogen, which is increasingly synthesized during the stationary phase (Velmurugan and Incharoensakdi 2018) and is stated to be rather a quick-response energy and carbon storage to cope with darkness (night), when its degradation may be the only available energy source for the strict phototrophic cells (Damrow et al. 2016; Koch and Forchhammer 2021). Furthermore, glycogen accumulation is crucial for phycobilisome degradation and assembly of the thylakoid membrane in presence of light (Velmurugan and Incharoensakdi 2018). It is not fully understood why cyanobacteria produce PHB and glycogen in parallel, as both storage compounds compete for the carbon pool (Wu et al. 2002; Rueda et al. 2020; Fritz et al. 2021). The glycogen synthesis path from CO_2_ starts with 3-phosphoglycerate, which can also be converted into pyruvate, then into acetyl-CoA and finally into PHB (Rueda et al. 2020). However, PHB and glycogen levels are independent of each other (Velmurugan and Incharoensakdi 2018; Koch et al. 2019). Glycogen concentrations in *Synechocystis* PCC6803 are stated to range from 18.5% (Velmurugan and Incharoensakdi 2018) up to 50% (per cell dry weight, CDW) (Luan et al. 2019). The concentration rises with cultivation and peaks at the mid-stationary phase (22.7% CDW), additionally it increases under nitrogen deficiency (36.8% CDW) and glucose addition (41.3% CDW) (Monshupanee and Incharoensakdi 2014). So does PHB, whose maxima are reported to be about 10–20% (CDW) in chlorotic cells and can be further increased by genetic engineering to 63% when cultivated in nitrogen- and phosphorous-depleted medium and to 81% when acetate is added (Koch et al. 2020). Initial concentrations of glycogen and PHB in *Synechocystis* PCC6803 are reported to be approximately 12% and 1%, respectively (Dutt and Srivastava 2018).

The experiments conducted herein aimed at evaluating to what extent *Synechocystis* *sp.* can survive and adapt to stepwise increasing salt concentrations and how biomass concentration changes, especially in regard to glycogen, PHB and pigments. For these purposes, growth and cell composition of three *Synechocystis* strains were monitored at stepwise increasing salt (NaCl) concentrations (0% to 8%).

## Material and Methods

### Strains and Media

The strains *Synechocystis* sp. PCC6803, *Synechocystis* cf. *salina* CCALA192 and an isolated wild-type *Synechocystis* strain, called IFA3, were used. PCC6803 was obtained from the Pasteur culture collection (FRA) and CCALA192 from the culture collection of autotrophic organisms (CZE). IFA3 was isolated in 2018 from a pond in Lower Austria and identified by sequencing its 16S-rDNA (see supplementary material).

The strains were cultivated in a mineral medium based on BG-11 (Rippka et al. 1979), whose nitrogen and phosphorous contents were adjusted to allow an early biomass growth until starvation of nitrogen and phosphorous initializes PHB production within a single cultivation stage. Nitrogen limitation is indicated by a typical color switch from blue-green to olive-green and orange. Composition mineral medium per liter: NaNO_3_: 0.45 g, Fe(NO_3_)_3_.9H_2_O: 0.025 g, MgSO_4_.7H_2_O: 0.10 g, CaCl_2_.2H_2_O: 0.60 g, Na_2_CO_3_: 0.20 g, K_2_HPO_4_: 0.08 g, trace element solution 1.50 mL. Composition trace element solution per liter H_3_BO_3_: 0.509 g, CuSO_4_.5H_2_O: 0.150 g, KI: 0.181 g, FeCl_3_.6H_2_O: 0.293 g, MnSO_4_.H_2_O: 0.296 g, Na_2_MoO_4_.2H_2_O: 0.082 g, NiSO_4_.6H_2_O: 0.275 g, Co(NO_3_)_2_.6H_2_O: 0.100 g, ZnSO_4_.7H_2_O: 0.490 g, KAl(SO_4_)_2_.12H_2_O: 0.395 g, KCr(SO_4_)_2_.12H_2_O: 0.470 g.

### Experimental setup

**Fig. 1:**
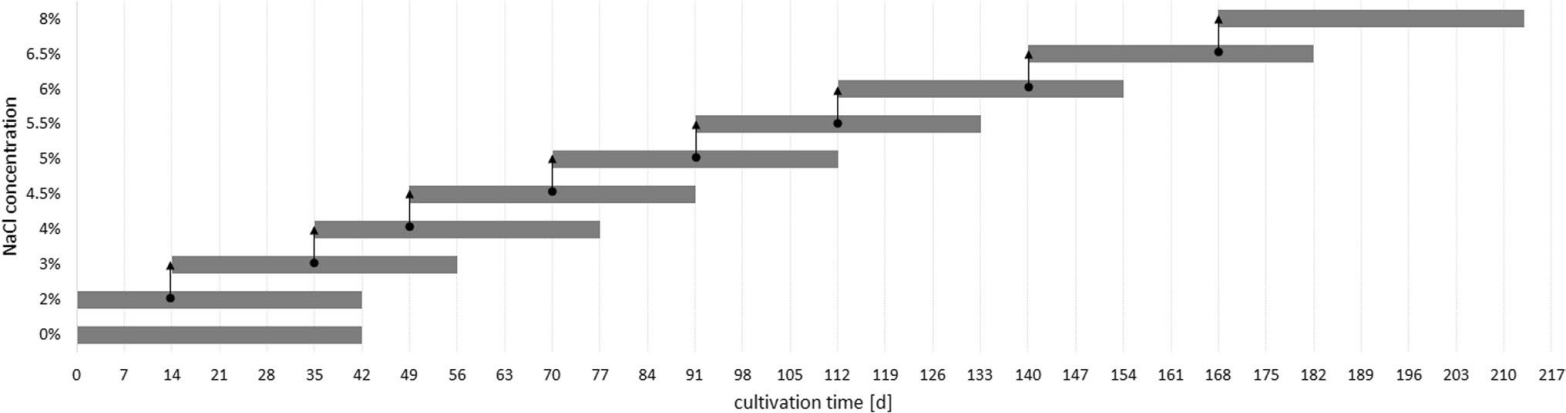
Time curve of the conducted experiments. Cells from the current stage were transferred to the next stage between weeks two and four (arrows)

In the herein conducted experiments, the salt (NaCl) concentration in the medium was stepwise increased. Cultivation of each stage was carried out in 200 mL mineral medium in 500-mL Erlenmeyer flasks in order to achieve a thin layer thickness of the cultures. The flasks were shaken manually two times per day. For cultivation ambient CO_2_ concentration (419 ppm – (McGee 2021)), 22 °C, 75.9 ± 9.5 µmol photons m^−2^ s^−1^ (metal halide lamp, Philips Master HPI-T Plus, 250 W) and a day/night cycle of 16/8 h were provided. The experiments were carried out as triplicates, each cultivation stage lasted 42 days. Cells were transferred from the current to the next stage between weeks two and four (see arrows in Fig. 1), depending on cell density (OD_750_). The OD_750_ at which the inoculum was taken was 2.20 ± 0.37 for PCC6803, 2.36 ± 0.39 for CCALA192 and 1.98 ± 0.74 for IFA3.

For the inoculation, about 40 mL of culture from each flask of the current stage were harvested (pooled from triplicates) and cell numbers were counted via Thoma chamber. All of the suspension was centrifuged gently at 800 × *g*, for 15 min, the cells were resuspended in the necessary amount of salt-containing mineral medium to get a cell density of 1.2–2 × 10^8^ mL^−1^. Thereof, 20 mL were transferred into 180 mL medium of the next step, resulting in 1.2–2 × 10^7^ cells mL^−1^. The NaCl concentration of the stages was set to 0% (w/v, reference), 2% (342 mM), 3% (513 mM), 4% (684 mM), 4.5% (770 mM), 5% (856 mM), 5.5% (941 mM), 6% (1027 mM), 6.5% (1112 mM) and 8% (1369 mM), respectively.

### Growth monitoring and Biomass analysis

Evaporation was compensated by adding the necessary amount of deionized water to each flask before samples were taken. Growth was monitored via optical density at 435 nm (OD_435_) and 750 nm (OD_750_) twice a week, also pH was measured in those samples. Once per week, the composition of the biomass was analyzed. Therefore, cells were separated from the cultivation media and washed with reverse osmosis water by centrifugation. Aliquots of wet biomass were stored at -20 °C until analysis of glycogen and pigments, and aliquots were dried at 105 °C for determination of cell dry weight (CDW) and PHB. The cell number of each sample was estimated by spectral flow cytometer (Cytek Aurora) where samples were individually washed and properly diluted by phosphate buffer (50 mM, pH 8).

For analyzing the PHB content, the dried pellet was digested with 200 µL concentrated (98%) sulfuric acid (H_2_SO_4_) for 30 min at 90 °C (adapted after (Karr et al. 1983)). In this step, PHB was converted to crotonic acid. Afterwards, the samples were filled to 10 mL with deionized water and prepared for HPLC (high-performance liquid chromatography) analysis (Agilent 1100; column: Transgenomic COREGEL 87H3; detector: Agilent 1100 RI).

The glycogen content of the cells was determined based on Koch et al. (2019). The pellet of 2 mL sample was resuspended in potassium hydroxide (KOH, 30% w/v) and incubated at 95 °C for 2 h. Ice cold ethanol (absolute) was added and the sample was incubated at -20 °C overnight to precipitate glycogen. Subsequently, the pellet was washed twice with ethanol (1^st^ wash: 70%, 2^nd^ wash: absolute), dried at 60 °C, resuspended in sodium acetate buffer (100 mM, pH 4.5), and digested with amyloglucosidase solution (0.62 U µL^−1^) at 55 °C and continuous shaking for 2 h. Carrez precipitation (method for removing turbidity such as colloids (Gänzle 2021)) was carried out to clarify the liquid and remove the enzyme. Finally, the sample was prepared for HPLC analysis (same configuration as for PHB analysis).

For analyzing contents of chlorophyll_a_ and total carotenoids (carotenoids) as well as c-phycocyanin (phycocyanin), the washed wet biomass was extracted with ethanol (absolute) (Lichtenthaler and Wellburn 1983; Ritchie 2006) and reverse osmosis water, respectively. Absorbances of the extracts were measured with a UV–Vis spectrometer (Shimadzu UV-1800). Pigment concentrations were calculated from the absorbance values of the extracts at certain wavelengths (chlorophyll_a_: 665 nm and 750 nm (Ritchie 2006); total carotenoids: 470 nm and 750 nm, modified after Lichtenthaler and Wellburn (1983); c-phycocyanin: 615 nm and 652 nm (Bennett and Bogorad 1973)) with equations (1-3):

1 $${Chlorophyll}_a\left[{mg\;L}^{-1}\right]=11.90355\;\ast\;\left(A_{665}-A_{750}\right)$$

2 $$Total\;carotenoids\left[{mg\;L}^{-1}\right]=\frac{1000\ast\left(A_{470}-A_{750}\right)-2.05\;\ast\;{chlorophyll}_a\left[{mg\;L}^{-1}\right]}{245}$$

3 $$C-phycocyanin\left[{mg\;L}^{-1}\right]=\frac{A_{615}-0.474\;\ast\;A_{652}}{5.34\;\ast\;1000}$$

Extraction-based pigment analysis was supplemented by their spectroscopic detection directly in cyanobacterial cell suspensions using diffusive transmission spectrophotometry. For this purpose, undiluted samples were measured in silica cuvettes on UV–Vis spectrophotometer Hitachi U-3900H with integrating sphere 60mm DIA at scan speed 600 nm min^−1^.

### Ultrastructure

For ultrastructural analysis, cyanobacterial cultures were centrifuged (4 min, 1000 *× g*). Samples for cryogenic scanning electron microscopy (cryo-SEM) were prepared by pipetting the concentrated pellet of cells on 6-mm Al carrier type A and closing with the flat side of type B. Samples were fixed using the high-pressure freezing method (EM ICE, Leica Microsystems) without using any cryo-protectant. Frozen samples were transferred into a cryo-vacuum preparation chamber (ACE600, Leica Microsystems) and then underwent freeze fracturing and sublimation at -95 °C for 7 min. No metal coating was applied. Following sublimation, cyanobacterial cells were observed in a scanning electron microscope (Magellan 400/L, FEI) equipped with a cryo stage at -120 °C using a 1–2 keV electron beam.

For transmission electron microscopy (TEM), the concentrated pellet of cells was pipetted on 3-mm Al carriers covered with 1% solution of lecithin in chloroform and fixed using the high-pressure freezing method (EM ICE, Leica Microsystems). Frozen samples were then transferred into a freeze-substitution unit (AFS2, Leica Microsystems). Substitution solution contained 1.5% OsO_4_ in acetone, the protocol used for the freeze-substitution was previously described in Kouřilová et al. (2021). After freeze substitution, samples were washed three times in acetone for 15 min each and gradually infiltrated with medium-hardness epoxy resin (Epoxy embedding medium, Sigma-Aldrich). Infiltration mixtures of epoxy resin and acetone in ratios 1:2, 1:1, 2:1, 1:0 were changed after 1 h. After the final exchange, samples were left in fresh pure resin under a vacuum in a desiccator overnight. Samples in fresh resin were cured using 62 °C heat for 48 h and cut to ultrathin sections on ultramicrotome (Ultracut UCT, Leica Microsystems) using a diamond knife (Diatome) with 45° cutting angle. Sections on 300 mesh copper grids were then stained using solutions of uranyl acetate and lead citrate and finally observed in transmission electron microscope JEOL 1010 using accelerating voltage 80 kV and images were digitally recorded by CCD camera Megaview III (Olympus).

## Results and Discussion

**Fig. 2:**
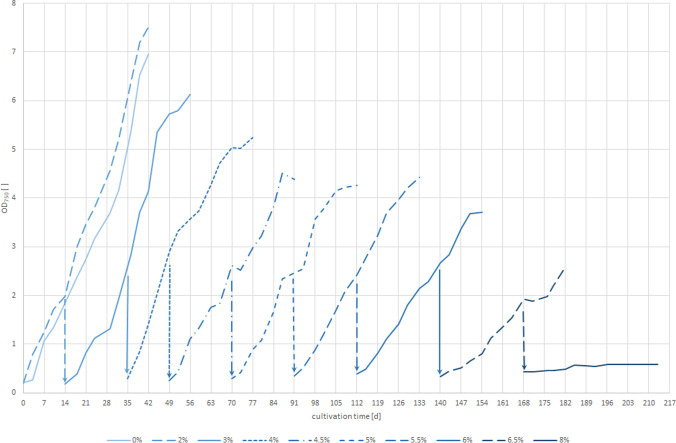
Growth curves of all experiments conducted with *Synechocystis* CCALA192. The solid lines show salt concentrations of 0%, 3%, 6% and 8% and are described in more detail in the text. The arrows indicate the inoculation of the next stage. n = 3

**Fig. 3:**
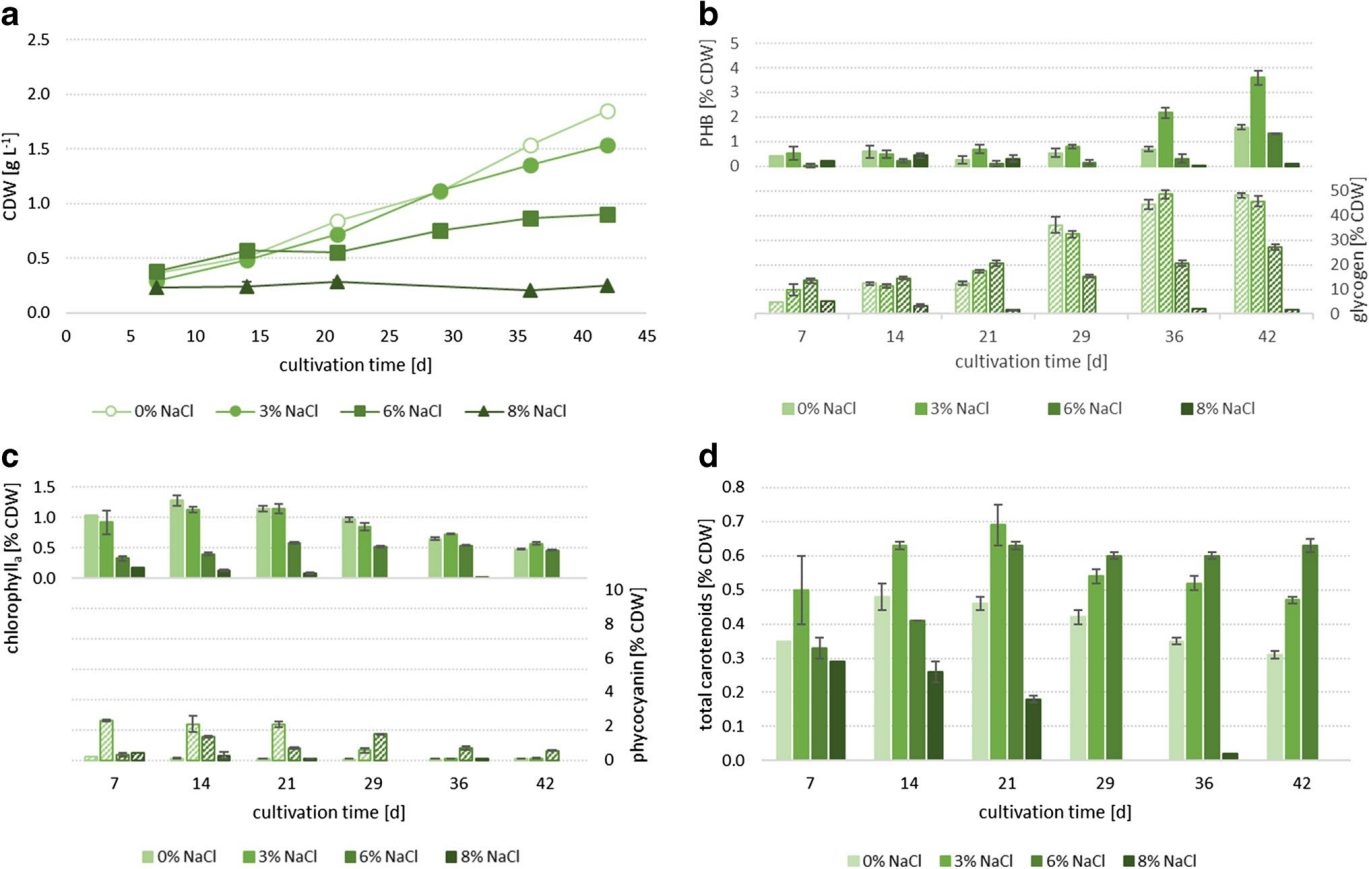
PCC6803 (**a**) growth, (**b**) PHB (filled columns) and glycogen (dashed columns), (**c**) chlorophyll_a_ (filled columns) and phycocyanin (dashed columns) as well as (**d**) carotenoids in 0%, 3%, 6% and 8% NaCl added to the medium; n = 3, error bars = standard deviation

**Fig. 4:**
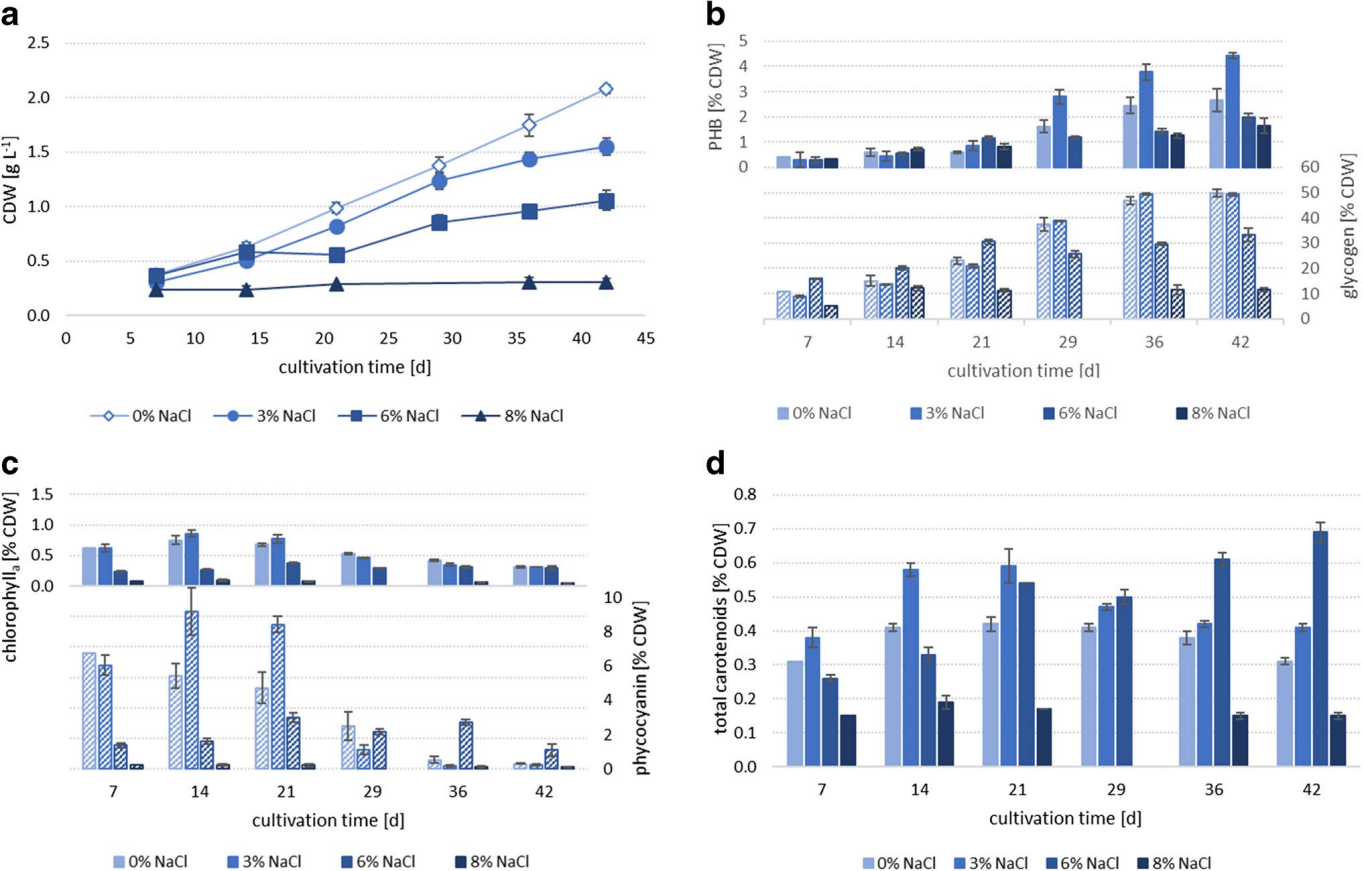
CCALA192 (**a**) growth, (**b**) PHB (filled columns) and glycogen (dashed columns), (**c**) chlorophyll_a_ (filled columns) and phycocyanin (dashed columns) as well as (**d**) carotenoids in 0%, 3%, 6% and 8% NaCl added to the medium; n = 3, error bars = standard deviation

**Fig. 5:**
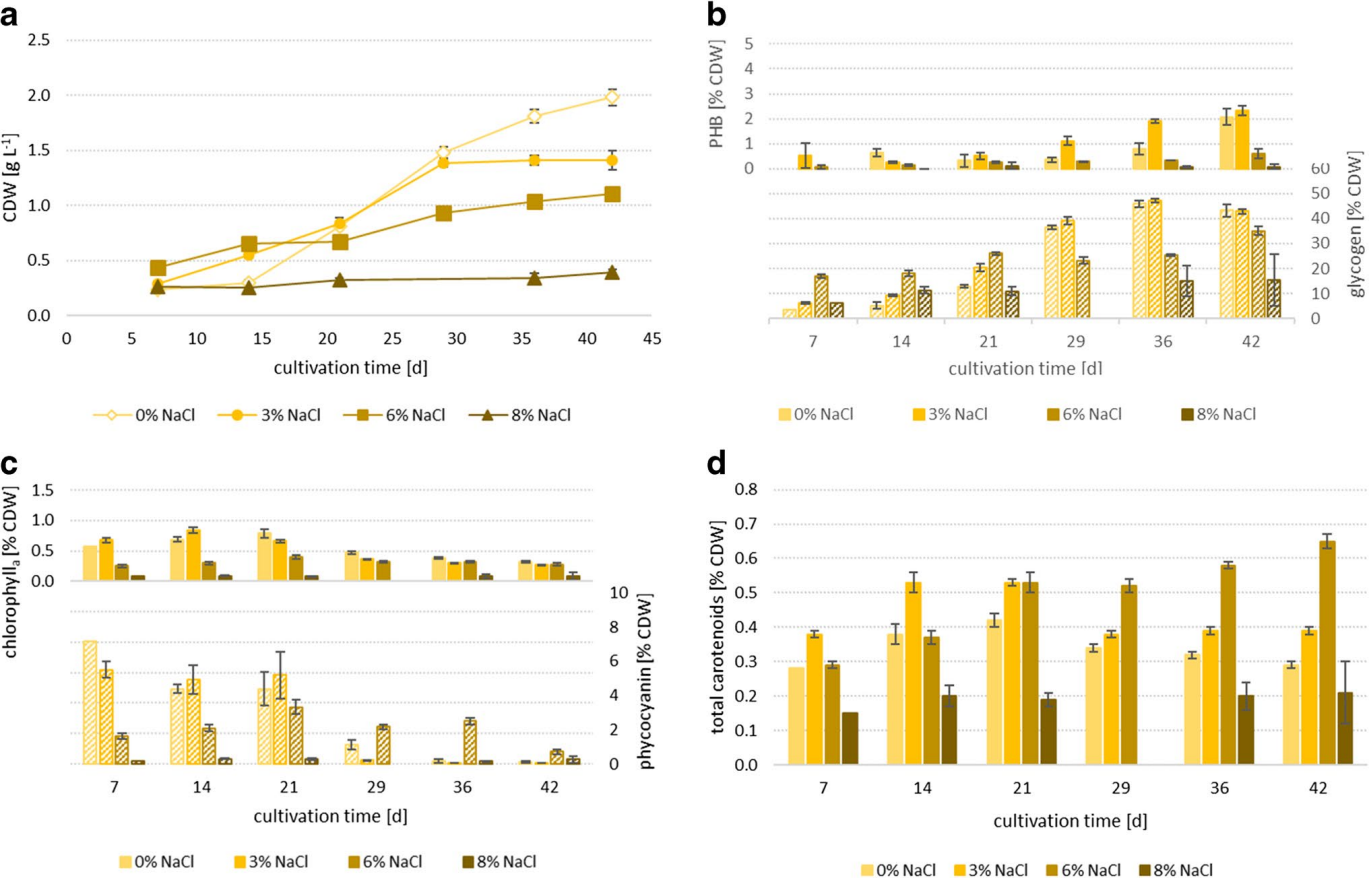
IFA3 (**a**) growth, (**b**) PHB (filled columns) and glycogen (dashed columns), (**c**) chlorophyll_a_ (filled columns) and phycocyanin (dashed columns) as well as (**d**) carotenoids in 0%, 3%, 6% and 8% NaCl added to the medium; n = 3, error bars = standard deviation

Three *Synechocystis* strains (PCC6803, CCALA192, IFA3) were exposed to stepwise increasing salt concentrations to evaluate how far they are able to adapt to increasing salt concentrations (from 0 to 8% w/v NaCl) and how biomass composition changes. The growth of CCALA192 (OD_750_) in all salt concentrations is shown as an example in Fig. 2 (those of PCC6803 and IFA3 are in the supplementary material). Salt concentrations of 0%, 3%, 6% and 8% (shown as solid lines) were chosen to illustrate the effect on growth and biomass composition (Figs. 3, 4, and 5). These stages represent no (0%, reference), moderate and high salt concentrations. The intermediate salt concentrations were necessary to gradually adapt the cells to the increasing salinity. The results of these stages do not show outstanding values nor do they provide different insights. Therefore, we refrained from presenting all data in the main section of this manuscript. The whole data (growth curves at OD_750_, OD_485_ [-], OD_750_ [-], pH [-], CDW [g L^−1^], PHB [mg L^−1^], glycogen [mg L^−^^1^], chlorophyll_a_ [mg L^−1^], phycocyanin [mg L^−1^], total carotenoids [mg L^−1^]) can be found in the supplementary material.

### Growth

The cell dry weight (CDW) of all the three *Synechocystis* strains (PCC6803: Fig. 3a, CCALA192: Fig. 4a, IFA3: Fig. 5a) was highest in the reference cultivations, without salt addition (0% NaCl). CCALA192 demonstrated the highest biomass concentrations (2.08 ± 0.04 g L^−1^) followed by IFA3 (1.98 ± 0.07 g L^−1^) and PCC6803 (1.85 ± 0.03 g L^−1^). For all three strains, it is clear that growth decreased with increasing salt concentrations and stagnated at a salt concentration of 8%. In the medium with 3% salt the final biomass concentrations of all three strains were comparable – PCC6803: 1.54 ± 0.02 g L^−1^, CCALA: 1.55 ± 0.08 g L^−1^, IFA3: 1.41 ± 0.09 g L^−1^. In the medium with 6% salt IFA3 showed the highest biomass concentrations (1.11 ± 0.05 g L^−1^), followed by CCALA192 (1.06 ± 0.09 g L^−1^) and PCC6803 (0.90 ± 0.01 g L^−1^). In 8% NaCl only IFA3 showed slight growth and reached 0.39 ± 0.03 g L^−1^ after a cultivation time of 42 days while both, CCALA192 and PCC6803, did not show any growth. Trends in cell numbers of cultures of CCALA192 and PCC6803 (42 days of cultivation), determined by flow cytometry (data not shown) and CDW, respectively, corresponded to each other.

As expected, the exposition of cyanobacterial cultures to stepwise increasing salinities in cultivation media partially inhibited the growth of the cells, with the inhibitory effect being more pronounced the higher the salt concentration in the culture medium. Velmurugan and Incharoensakdi (2018) stated that growth of moderately halotolerant cyanobacteria *Synechocystis* is hardly affected up to 100 mM (0.6%) NaCl and results in a biomass concentration of 1.4 g L^−1^. With increasing salt concentrations, biomass concentrations declined from 1.3 g L^−1^ (200 mM = 1.2% NaCl) to 1.1 g L^−1^ (300 mM = 1.8% NaCl) (day 20). These values (achieved at 100 µmol photons m^−2^ s^−1^, 28 °C, in BG-11 medium) are within the range of those achieved by our experiments (0.61 to 0.96 g L^−1^ at 2% NaCl and day 21, 1.55 to 2.04 g L^−1^ at 2% NaCl and day 42). Also, achieved results about biomass were confirmed by data from flow cytometer, i.e., cultures with increasing salt concentrations demonstrated decreasing trend also in cell number (data not shown).

### PHB

All three strains produced PHB. The cellular concentration of PHB increased over cultivation time (PCC6803: Fig. 3b, CCALA192: Fig. 4b, IFA3: Fig. 5b). In the reference cultivations (0% NaCl), lowest PHB concentrations were achieved. The PHB content increased up to a certain salt concentration, which was individually different for each strain and was then decreasing when salinity further rose. The highest PHB concentration (6.98 ± 0.10% CDW) was achieved by CCALA192 in 4% salt after 42 days of cultivation, followed by PCC6803 (3.60 ± 0.28% CDW) in 3% and IFA3 (3.27 ± 0.8% CDW) in 2%, after 42 and 36 days, respectively. The same trend is visible when comparing PHB concentrations of the strains after the same cultivation time (42 days) in 3% salt — CCALA192: 4.43 ± 0.10% CDW, PCC8603: 3.60 ± 0.28% CDW, IFA3: 2.33 ± 0.19% CDW. At all the salt concentrations, CCALA192 accumulated higher PHB amounts than the other two strains.

The PHB contents of all the three strains in the reference cultivations (0% NaCl) were by far lower (0.21 to 0.61% CDW, day 21) than reported for *Synechocystis* PCC6803 (about 2.5% CDW, day 20, at 100 µmol photons m^−2^ s^−1^, 28 °C, in BG-11 medium) (Velmurugan and Incharoensakdi 2018) and for nitrogen depleted cells (about 15–16.5% CDW, day 12, at 150 µmol photons m^−2^ s^−1^, 30 °C, in BG-11 medium + 0.4% (v/v) acetate) (Dutt and Srivastava 2018). PHB concentration increased with increasing cultivation time but did not exceed 2% CDW in PCC6803, when cultivation was stopped on day 42. Reasons could be the lower temperature and light intensity in our experiments lead to lower growth compared to literature. And most likely that nitrogen deprivation occurred later than day 20 or 12, due to the used medium, which we adapted for single-stage cultivation. Furthermore, no organic carbon was added as described by Dutt and Srivastava (2018). IFA3 and CCALA192 contained 2.1% and 2.7% CDW at the end of cultivation.

### Glycogen

Glycogen concentrations (PCC6803: Fig. 3b, CCALA192: Fig. 4b, IFA3: Fig. 5b) were — comparable to PHB concentrations — increasing in all the three strains with increasing cultivation time. Another similarity to PHB accumulation was that CCALA192 also had the highest glycogen concentrations but in contrast in 3% NaCl. The second highest glycogen concentration was achieved by PCC6803, followed by IFA3. In all the strains the highest glycogen levels were accumulated in 3% NaCl — CCALA192 (49.43 ± 0.58% CDW) after 42, by PCC6803 (48.60 ± 1.82% CDW) and IFA3 (47.24 ± 0.73% CDW) after 35 days. With further increasing salt concentrations glycogen levels decreased. This effect is clearly visible later in cultivation (> day 29). The glycogen concentrations in the reference cultivations (0% NaCl) obtained 12.45 ± 0.66% CDW (PCC6803), 23.04 ± 1.21% CDW (CCALA192) and 12.80 ± 0.48% CDW (IFA3) on day 21. By increasing salinity to 6%, glycogen concentrations of 20.67 ± 1.25% CDW (PCC6803), 20.93 ± 0.63% CDW (CCALA192) and 20.26 ± 1.48% CDW (IFA3) were achieved on day 21, while on day 42 it had just increased toward 27.24 ± 1.18% CDW, 33.46 ± 2.63% CDW and 35.05 ± 1.66% CDW in PCC6803, CCALA192 and IFA3, respectively.

In contrast to PHB, glycogen levels were high at low salt concentrations. PHB and glycogen in common increased up to certain salt concentrations (e.g., 3% NaCl for glycogen and 4% NaCl for PHB, both in CCALA192) and then declined. For glycogen, higher concentrations are stated to be produced in 4% NaCl than in 0% (Pade et al. 2017). When comparing reference cultivations (0% NaCl) of *Synechocystis* PCC6803 higher glycogen values were achieved in the literature — 24.1% CDW within 21 days (Velmurugan and Incharoensakdi 2018) — than compared to herein cultivated PCC6803 and IFA3 (12.45 ± 0.66% CDW and 12.80 ± 0.48% CDW, respectively, in reference cultivations (0% NaCl), day 21). The values obtained by CCALA192 (23.04 ± 1.21% CDW, reference cultivations (0% NaCl), day 21) were within the range. By genetic modifications (glycogen overexpression combined with *phaA*-knockout), glycogen concentrations were further increased to 38.8% CDW on day 20 and 40.4% CDW on day 25 (Velmurugan and Incharoensakdi 2018). These values were twice as high as those achieved in the reference cultivations (0% NaCl) on day 21 but within the range obtained at 3% NaCl after 42 cultivation days (PCC6803: 45.84 ± 2.02% CDW; CCALA192: 49.43 ± 0.58% CDW; IFA3: 42.85 ± 1.08% CDW). Glycogen concentrations can be even further enhanced by nitrogen deprivation (to about 42% CDW, after 12 days). Besides that, glycogen concentrations especially at the beginning of cultivation are elevated when cells are pre-grown under photomixotrophic conditions (slightly below 60% CDW at the beginning of cultivation and slightly above 50% CDW after 12 days) compared to phototrophic conditions (about 3% CDW at the beginning and slightly over 40% CDW after 12 days) (Dutt and Srivastava 2018).

### Salt Stress and its Effect on the Accumulation of PHB and Glycogen

The average PHB-to-glycogen ratios over the cultivation time in 3% salt were comparable between all three strains (1:21 for CCALA192, 1:24 for PCC6803 and 1:27 for IFA3), suggesting that PHB and glycogen synthesis pathways in all three strains were about equally strong at moderate salt concentrations. With increasing or decreasing salt concentrations this ratio changed. PCC6803 produced 40 and 147 times more and IFA3 55 and 129 times more glycogen than PHB at 0% and 6% NaCl, respectively. Only glycogen accumulation in CCALA192 stood rather constant at 1:25 and 1:29 in 0% and 6% NaCl. Summarized, all three strains accumulated more glycogen than PHB, but ratios changed with changing salinity. The fact that medium salinity substantially affects PHB and glycogen content in cyanobacteria is not only of fundamental interest but it might be also of biotechnological importance since NaCl addition could be used as a tool to enhance PHB synthesis; nevertheless, the salt should be applied at later stages of cultivation to not inhibit the growth of the culture (since PHB is an intracellular metabolite and high CDW values are the necessary prerequisite for high PHB titers).

The fact that fractions of both storage compounds are lower in cultures exposed to high osmotic pressures can be attributed to inhibition of the photosynthetic system resulting in a decreased amount of carbon and energy for storage polymers synthesis (see further in the text). Nevertheless, it is also likely that carbon and energy expenses of stress-response against osmotic pressure (excretion of Na^+^, production of compatible solutes, modulation of gene expression etc.) also contributed to decreased amounts of storage polymers in the cultures. Hence, if NaCl should be considered as a tool to improve PHB production, its concentration must be wisely chosen and precisely controlled since above a certain value it has a negative impact on PHB synthesis.

### Spectroscopic Identification of Pigments in Cell Suspensions of *Synechocystis* strains PCC6803 and CCALA192

**Fig. 6:**
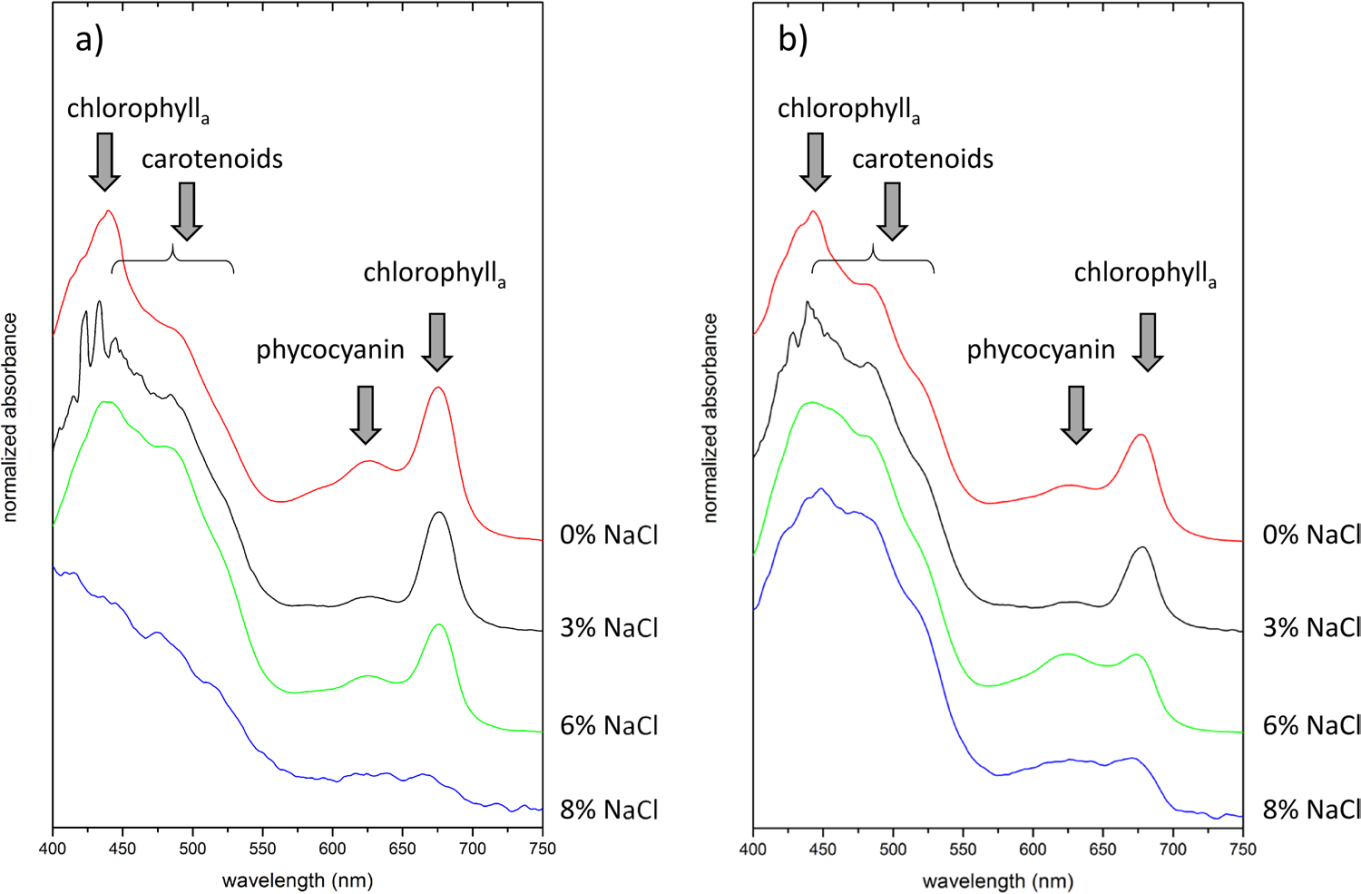
VIS absorption spectra (400–750 nm) of cultures after 42 days of cultivation in 0%, 3%, 6% and 8% NaCl added into the medium a) PCC6803 b) CCALA192. Spectra were obtained by diffusive transmission spectrophotometry

To identify and analyze pigments directly in the cultivated culture, we used a less common spectroscopic method of diffusive transmission spectrophotometry that allows the determination of UV-VIS absorption spectra for highly turbid samples. All the measured cultures were at the same stage of cultivation (42 days). As can be seen from the comparison of corresponding pigment contents shown in Fig. 3c/d, Fig. 4c/d, Fig. 5c/d and changes in the spectral fingerprints of the pigments in Fig. 6, there is a good agreement in the observed effect of the salt present in cultivation media on the changes in pigments composition.

### Quantification of Pigments obtained by Extraction 

#### Chlorophyll_a_

In all the three *Synechocystis* strains, the chlorophyll_a_ contents rose until day 14 (0% and 3% NaCl, PCC6803, CCALA192, IFA3) or 21 (6% NaCl, PCC6803, CCALA192, IFA3) and then decreased with increasing cultivation time. Additionally, chlorophyll_a_ tends to decrease at higher salt stress levels (PCC6803: Fig. 3c, CCALA192: Fig. 4c, IFA3: Fig. 5c). Chlorophyll_a_ was highest in PCC6803 (1.34 ± 0.02% CDW) in 2% NaCl on day 14, compared to IFA3 and CCALA192, whose chlorophyll_a_ levels also peaked (1.01 ± 0.03% CDW and 0.93 ± 0.06% CDW, respectively) in 2% NaCl on day 14.

Concerning cultivation time, an increasing trend of chloropyll_a_ in *Synechocystis* PCC6803 and engineered strains (glycogen overexpression, *phaA*-knockout) thereof is reported for growth in salt free BG-11 medium from day 10 to 15, followed by a decrease to day 20 (Velmurugan and Incharoensakdi 2018). This trend could be confirmed by PCC6803, CCALA192 and IFA3 whose chlorophyll_a_ content in reference cultivations (0% NaCl) rose until day 14 or 21 and then declined until the end of cultivation.

The results obtained for chlorophyll_a_ at different salt concentrations were comparable with those collected by Sudhir and Murthy (2004), who stated that at 342 mM (2%) NaCl the chlorophyll_a_ content in *Synechocystis* PCC6803 increased, while at 684 mM (4%) or 1026 mM (6%) it sharply decreased.

As the chlorophyll_a_ content decreased, growth also decreased but did not stop completely. The decrease in chlorophyll_a _was not only observed with cultivation time, but also with increasing salt concentration. Higher salt concentrations led to a faster decline in chlorophyll_a_. However, even low chlorophyll_a_ concentrations were sufficient for *Synechocystis* to continue growing. In case of nitrogen chlorosis, phycocyanin (peak at 630 nm) and to a lesser extent chlorophyll_a_ (peak at 680 nm) gradually decreased with increasing cultivation time (Krasikov et al. 2012) and growth stopped (Klotz et al. 2016).

In glycogen overexpressing strains, chloropyll_a_ contents were lower compared to the wild type and *phaA*-knockout strain, respectively. Despite low chlorophyll_a_ contents, oxygen evolution rate was highest in the combined strain (glycogen overexpression and *phaA*-knockout), which enhanced overall biomass concentration (Velmurugan and Incharoensakdi 2018). Similar patterns can be seen when comparing chlorophyll_a_ and glycogen contents of PCC6803 and CCALA192. Chlorophyll_a_ contents of PCC6803 were generally higher, while glycogen levels and growth were slightly lower than in CCALA192. This suggests that the efficiency or the content of PSII (photosystem II, to which oxygen evolution is connected (Allakhverdiev and Murata 2008)) in PCC6803 is rather low compared to the other two strains.

#### Phycocyanin

Phycocyanin showed similar tendencies as chloropyll_a_; its contents rather decreased with increasing cultivation time (PCC6803: from day 7 at 3%, from day 29 at 6%; CCALA192: from day 7 at 0%, from day 14 at 3%, from day 21 at 6%; IFA3: from day 7 at 0% and 3%, from day 21 at 6%) (PCC6803: Fig. 3c, CCALA192: Fig. 4c, IFA3: Fig. 5c). Related to salt concentrations, the picture is more complex: The highest phycocyanin levels were reached by CCALA192 (9.17 ± 1.38% CDW) in 3% NaCl at day 14 and by IFA3 (7.17% CDW) in 0% at day 7. PCC6803 reached a maximum of only 2.34 ± 0.05% CDW in 3% NaCl on day 7. In 4% NaCl phycocyanin concentrations were comparably low — the maximum was 2.32 ± 0.11% CDW in CCALA192 at day 14. At higher salinities, phycocyanin then rose approximately until mid of cultivation (day 21, day 28) and decreased again. At salinities equal to or higher than 6% NaCl, phycocyanin levels did hardly change during cultivation. Especially the strains CCALA192 and IFA3 produced plenty of phycobiliproteins in contrast to PCC6803, but with a much more pronounced difference in salt free medium (shown in Figs. 3c, 4c and 5c). Our results show that the phycocyanin content is highly dependent upon the cyanobacterial strain, cultivation and salt concentration of the cultivation medium.

Sudhir and Murthy (2004) as well as Hagemann et al. (1999) mentioned, as also demonstrated herein, that the phycocyanin content decreases under salt stress. This in turn reduces the energy transfer to PSII (Sudhir and Murthy 2004), since phycocyanin belongs to the phycobiliproteins, which form a photosynthetic antenna and lead electrons to chlorophyll_a_ in the photosynthetic membrane (thylakoid membrane) (MacColl and Guard-Friar 2018). However, according to Schubert et al. (1993) cells grown under suboptimal conditions use pigments for photosynthesis with higher efficiency. Not only elevated salt concentrations but nitrogen limitation also results in the degradation of phycocyanin, because phycobiliproteins are also nitrogen storage molecules. While the cells break down phycobiliproteins, they stop growing after a final cell division and at the same time accumulate CO_2_ fixation products such as glycogen and PHB. Then, the chloropyll_a_ content decreases and chlorotic cells turn toward a dormant state (Krasikov et al. 2012; Klotz et al. 2016; Velmurugan and Incharoensakdi 2018).

We observed degradation of phycocyanin during cultivation in salt concentrations less than or equal to 5.5% NaCl. At further increasing salinity, phycocyanin concentrations did hardly change during cultivation. Phycocyanin concentrations in combination with CDWs (which were highest at lowest salinity) indicate that at low to moderate salt concentrations (0% to 3%), nitrogen limitation and therewith phycocyanin degradation occurred due to growth. This was caused by adjusted nitrogen and phosphorous contents in the medium, which was used to allow biomass growth and PHB accumulation in single-stage cultivation. At higher salt concentrations (684 mM = 4% NaCl), more proteins were needed to cope with the stress conditions (Hagemann et al. 1999; Fulda et al. 2000; Huang et al. 2006; Tanniche et al. 2020). For this elevated protein production, more nitrogen was bound, which in turn led to nitrogen limitation and consequently resulted in declining or low but stable phycocyanin concentrations (at salinities higher than or equal to 6% NaCl). In the plasma membrane of *Synechocystis*, mainly substrate-binding proteins of hypothetical proteins and ABC transporters are enhanced during salt stress (684 mM = 4% NaCl), which are suggested to be involved in thylakoid membrane function and to protect PSII (Huang et al. 2006). Proteins of the periplasm are involved in the generation and modification of external cell layers (e.g., slime), which also seem to be affected by salt stress to build an enhanced diffusion barrier and reduce ion influx (Fulda et al. 2000). As indicated by cryo-SEM images (Fig. 8), PCC6803 produced more slime than CCALA192, yet CCALA192 appeared to be more resistant to salt stress, yet PCC6803 may have produced more slime precisely because of less salt resistance.

In PCC6803, the chlorophyll_a_ content was higher and phycocyanin was by far lower compared to the other strains. Therefore, more photons in the range of chlorophyll_a_ (440 nm and 678 nm (Luimstra et al. 2019)) can be absorbed, but the spectral range of phycocyanin (maxima at 615 nm (MacColl and Guard-Friar 2018)) is almost completely missing and may lead to a lower energy yield. All together this might indicate that the accumulation of glycogen needs less energy compared to PHB. Furthermore, glycogen is stated to be quick-response storage (Koch and Forchhammer 2021) to survive short-term stress conditions as darkness and acclimation to nutrient depletion (Damrow et al. 2016). So, it seems that PCC6803 would rather invest in quick-response storage. This would also explain the slightly slower growth of PCC6803 at higher salt concentrations. IFA3 achieved chlorophyll_a_ concentrations comparable to those of CCALA192 but lower phycocyanin concentrations and therewith probably suffered from lower energy yields than CCALA192, which was maybe comparable to PCC6803. Growth of IFA3 and PCC6803 was comparable at 0% NaCl, slightly lower at 3% NaCl but higher at 6% NaCl, where phycocyanin and glycogen concentrations were higher in IFA3 and only slowly degraded. This and the still observable growth of IFA3 at 8% NaCl may suggest that this strain has additional mechanisms to cope with salt stress.

#### Carotenoids

Carotenoids (PCC6803: Fig. 3d, CCALA192: Fig. 4d, IFA3: Fig. 5d) tended to decrease with ongoing cultivation time at least up to a concentration of 3% NaCl. At salinities equal to or higher than 5%, carotenoids increased with cultivation time until day 21, approximately, and then stayed rather stable until the end of cultivation, being a strong contrast to chlorophyll_a_ and phycocyanin levels. Highest carotenoid concentrations were reached by PCC6803 (0.65 ± 0.02% CDW) in 6.5% NaCl on day 28, followed by IFA3 (0.65 ± 0.02% CDW) and CCALA192 (0.69 ± 0.03% CDW) in 6% NaCl at the end of cultivation (day 42).

At salt concentrations equal to or higher than 5.5% carotenoid levels rose with ongoing cultivation time and stayed then rather constant. This trend is confirmed by Sudhir and Murthy (2004), who stated that carotenoids increase when high (1026 mM, 6%) NaCl concentrations occur. Since carotenoid contents were high and those of PHB were rather low at high salt concentrations, we assume that *Synechocystis* cells protect themselves against damages through salt stress by directing carbon toward carotenoids instead of PHB. Carotenoids are light-harvesting and photoprotective pigments (Mills et al. 2020) and are assumed to shade chlorophyll_a_ and consequently reduce the number of photons available for absorption by chlorophyll_a_ (Schubert et al. 1993; Sudhir and Murthy 2004). Thus, another stress factor — irradiation — can be reduced. Rezayian et al. (2019) agree and state that β-carotene, a lipophilic and non-enzymatic antioxidant, protects the light-harvesting pigments against photochemical damage. The combination of salt and light stress inactivates PSII very quickly since salt stress prevents the repair of photodamaged PSII (Allakhverdiev et al. 2002). Additionally, carotenoids are essential for membrane integrity and thylakoid organization (Mills et al. 2020). Besides that, β-carotene harvests light (450–520 nm) for PSI (photosystem I) (Stamatakis et al. 2014) and is important for the cell to gain energy.

### Salt Stress and its Effect on the Photosystems

Allakhverdiev and Murata (2008) state that the activity of PSII (photosystem II) and PSI (photosystem I) is reduced within a few minutes after exposing cyanobacterial cells to salt stress. Depending on the duration of the stress, the activity can be fully restored, when salt concentrations and the therewith connected osmotic levels are reversed back to normal. Compared to osmotic stress induced by 500 mM sorbitol, which decreases the cytoplasmic volume to 30–50% of the initial volume within minutes, salt stress affects the cell volume less. Less water leaves the cell but Na^+^ and Cl^−^ ions enter through K^+^(Na^+^) and Cl^−^ channels, causing increased intracellular concentration of Na^+^ and Cl^−^. Na^+^/H^+^ antiporters regulate intracellular Na^+^ concentrations but this active ion export is energy demanding (Hagemann et al. 1999). Increased Na^+^/Cl^−^ concentrations lead to dissociation of extrinsic proteins of the photosystems, which gets irreversibly damaged within hours. Weak light (70 µmol photons m^−2^ s^−1^, being within the intensity range used for our experiments) restores the activity of PSII, PSI and Na^+^/H^+^ antiporters and therefore proves important for the tolerance of photosystems to salt stress (Allakhverdiev and Murata 2008).

Salt stress impairs the photosynthesis rate, affects growth rate (demonstrated in Figs. 3a, 4a, and 5a) and inhibits the oxygen evolution activity, which is mediated with PSII (Sudhir and Murthy 2004). But in contrast, the content of PSI as well as its activity and therefore cyclic electron flow around PSI are enhanced in salt-loaded *Synechocystis* sp. PCC6803. That in turn leads to higher ATP production (photophosphorylation), increases respiratory oxygen consumption, which is connected to enhanced activity and content of cytochrome oxidase as well as increased CO_2_ fixation rate (Hagemann et al. 1999; Sudhir and Murthy 2004).

Increased CO_2_ fixation rate was reflected by increased glycogen and especially PHB concentrations (Figs. 3b, 4b and 5b — 3% NaCl), both are the main CO_2_-fixation products (Klotz et al. 2016; Velmurugan and Incharoensakdi 2018). Glycogen is accumulated around thylakoid membranes. Glycogen (Velmurugan and Incharoensakdi 2018) and PHB act as electron sinks and contribute to the maintenance of the desired redox potential by eliminating an excess of reducing equivalents, thereby protecting cells from intracellular redox stress (Koch and Forchhammer 2021). If PHB cannot be produced (*pha*A-knockout), *Synechocystis* redirects the carbon flow toward glycogen synthesis. Whereas glycogen-overexpression hardly affects PHB content, suggesting that PHB synthesis is independently regulated (Velmurugan and Incharoensakdi 2018) and is not reduced as long as the necessary synthesis energy is available. In contrast to this, it is also reported that during nitrogen starvation *Synechocystis* sp. PCC 6803 cells produce PHB from glycogen (Koch et al. 2019). As described in the literature and demonstrated in the experiments performed, both the growth and accumulation of carbon storage compounds decreased with increasing salt concentration. Therefore, it is assumed that above a certain salt concentration (which depends on the strain), even under low light conditions (which restores the activity of photosystems and antiporters) increasingly more energy is required for countermeasures to salt stress. Thus, less capacity is available for carbon storage compounds.

### Ultrastructure

**Fig. 7:**
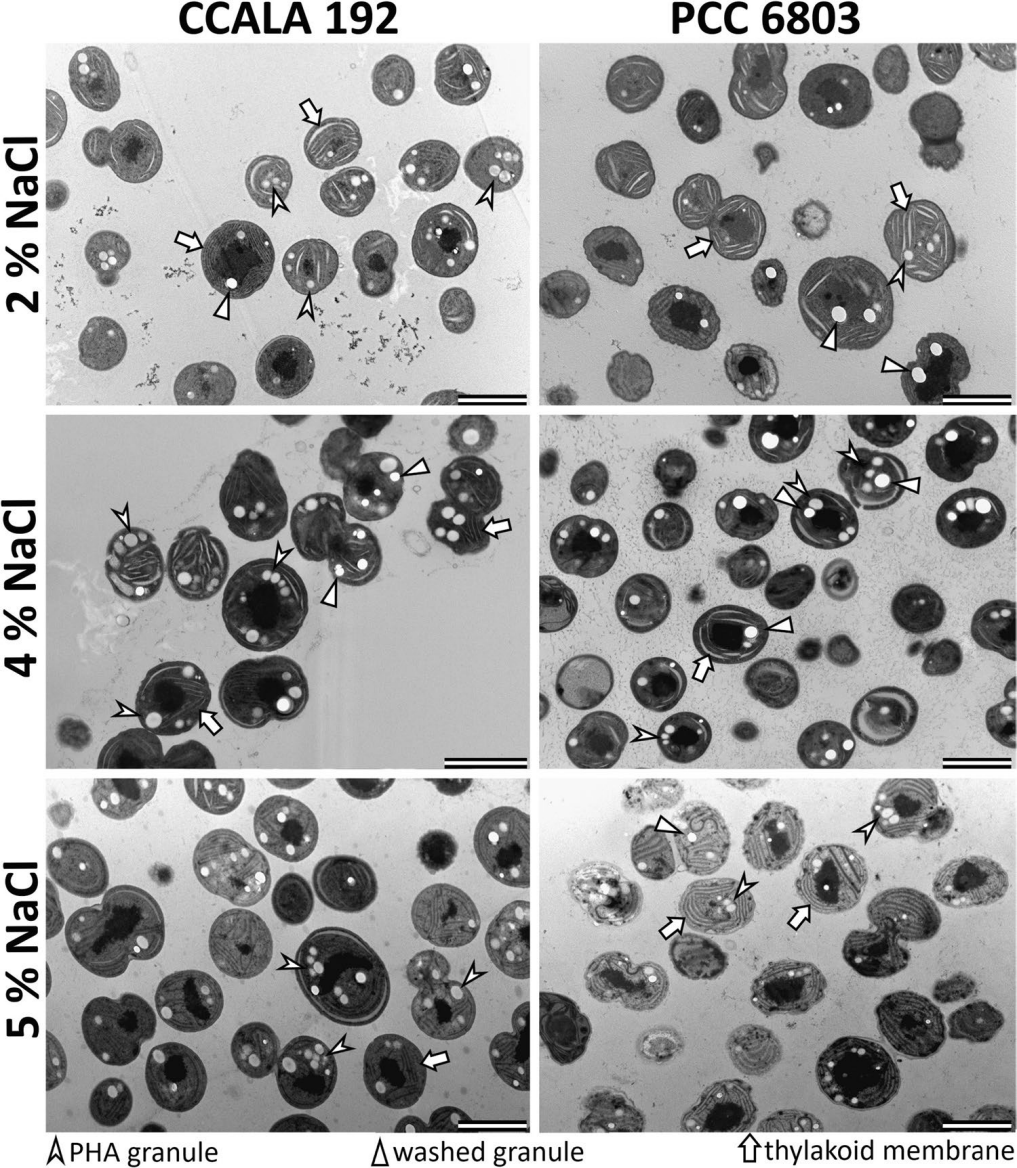
TEM images of cyanobacterial cells CCALA192 and PCC6803 exposed to concentration of 2%, 4% and 5% of NaCl, scalebar 2 µm

Focusing on strain CCALA192 (Fig. 7 left), it was possible to count a higher number of PHB granules at moderate and high salt concentrations as well as a higher presence of dense structures in the center of cells possibly containing compacted DNA. There were no significant effects of the rising salt concentrations on the cellular ultrastructure. Focusing on strain PCC6803 (Fig. 7 right), it was possible to observe very dense-looking cells at the intermediate salt concentration in contrast with very pale, visibly more damaged, cells at high salt concentration. It was also possible to see a higher presence of the dense structures in the middle of the cells, just as in the other imaged strain. The PHB granules were smaller in PCC6803 at 5% NaCl compared to lower concentrations supporting the quantitative PHB analysis.

**Fig. 8:**
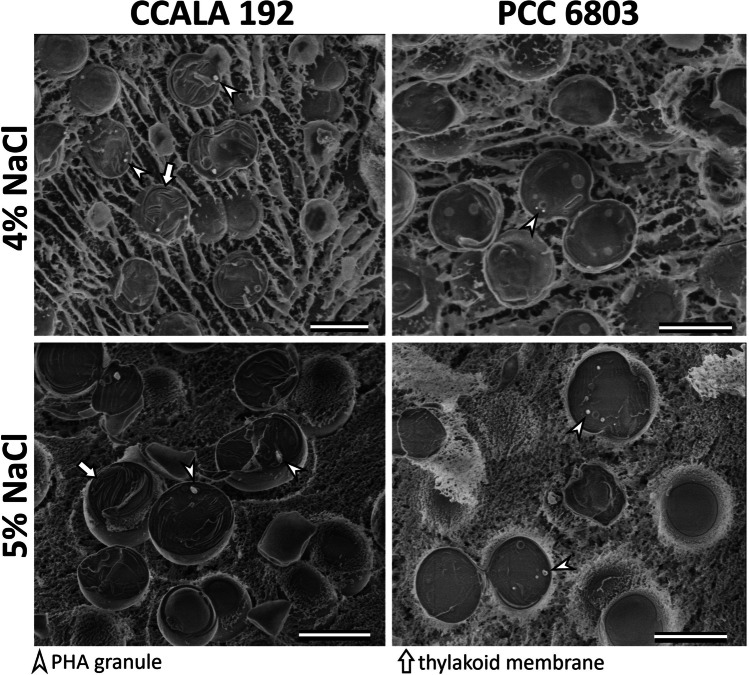
Cryo-SEM images of cyanobacterial cells CCALA192 and PCC6803 exposed to concentration of 4% and 5% of NaCl, scalebar 2 µm

Cryofixation enables to fix cells without the need for any chemical fixatives and then to observe cells in their most native state (Fig. 8). The images show PHA granules sticking out of fractured cells since PHB is elastic even at temperatures of liquid nitrogen as previously described in Obruča et al. (2016). Also, after freeze-fracture and sublimation, it was possible to observe thylakoid membranes in cells as well as a mucous layer surrounding the cells in figures showing 5% of NaCl concentration. The slime produced by cyanobacteria can be observed in cryo-SEM thanks to avoiding washing the cells with chemical fixatives, while in TEM the slime could have been washed away with acetone after freeze-substitution. In Fig. 8 at 4% salt concentration, some crystallization occurred during freezing in the ice surrounding the cells which, however, did not damage the cells. This artifact can be observed as holes in the ice elongated in the same direction. The successfulness of the fixation of the cells using cryogenic methods depends on the thickness of the samples, the amount of water in the sample (Hrubanova et al 2018) and also on the osmolarity of the solution surrounding the cells or usage of cryoprotectants (McDonald 2007). Since higher concentrations of salt in media can serve as a cryoprotectant (Husseini et al. 2006), it is possible that the samples in figures showing 5% NaCl concentration could have been better protected against the formation of freezing artifacts.

## Conclusion

All three cultivated *Synechocystis* strains succeeded in adapting to increasing salt concentrations. However, increasing salt concentrations affected not only the growth and accumulation of carbon storage compounds (glycogen and PHB), but also pigment composition. Pigments could also be analyzed directly from the samples without prior extraction and their absorption peaks could be compared. Low to moderate salt concentrations (3%) increased both PHB accumulation and chlorophyll_a_ and phycocyanin content. It is hypothesized that at high salt concentrations (6%), cells require more energy for countermeasures against salt stress and less is available for building carbon storage compounds as effect less glycogen and PHB are formed.

Concerning the survival in arid or semi-arid regions, strain CCALA192 would have the best chance as it was found to be more resistant or tolerant to salt. It also grew faster at higher salt concentrations and accumulated more glycogen and PHB, while maintaining its phycocyanin content and thus its photosynthetic activity. Therefore, and because its biomass and PHB production resists unfavorable conditions, this strain is attractive for growth experiments or PHB production at a larger scale. In addition to choosing a promising production strain, NaCl could also be considered as a tool to enhance PHB production. However, the concentration needs to be chosen wisely and controlled closely. Cultivation in salty liquids would not only allow the use of seawater but also of saline wastewater or residual streams (retentate from reverse osmosis plants, fermentation residues with elevated salt concentration, etc.) for the production of PHB, which would conserve resources or use them sustainably.

## Data Availability

All data generated or analyzed during this study are included in this published article [and its supplementary information files].
